# Dynamics of Local Temperature in the Fingertips After the Cuff Occlusion Test: Infrared Diagnosis of Adaptation Reserves to Hypoxia and Assessment of Survivability of Victims at Massive Blood Loss

**DOI:** 10.31083/j.rcm2305174

**Published:** 2022-05-13

**Authors:** Aleksandr Urakov, Natalya Urakova, Anton Kasatkin, Aleksandr Samorodov, Valentin Pavlov

**Affiliations:** ^1^Department of General and Clinical Pharmacology, Izhevsk State Medical Academy, 426034 Izhevsk, Udmurt Republic, Russia; ^2^Department of Modeling and Synthesis of Technological Structures, Institute of Mechanics, Udmurt Federal Research Center, 426067 Izhevsk, Udmurt Republic, Russia; ^3^Department of Obstetrics and Gynecology, Izhevsk State Medical Academy, 426034 Izhevsk, Udmurt Republic, Russia; ^4^Department of Pharmacology, Bashkir State Medical University, 450000 Ufa, Republic of Bashkortostan, Russia; ^5^Department of Urology, Bashkir State Medical University, 450000 Ufa, Republic of Bashkortostan, Russia

**Keywords:** hemorrhage, hypoxia, resistance, adaptation, fingertips, local hypothermia, infrared imaging temperature

## Abstract

**Background:**

Since changes in the tone and size of the lumen of peripheral blood vessels with massive blood loss are part of the mechanism of adaptation to hypoxia, which automatically changes the flow of warm blood to the fingertips, it was assumed that infrared thermography of the fingertips can reveal the dynamics of heat release in them, reflecting the reactivity of peripheral blood vessels and adaptation to hypoxia. It was assumed that the cuff occlusion test (COT) would assess the available reserves of adaptation to hypoxia and improve the accuracy of resistance to hypoxia and the prognosis of survival in massive blood loss.

**Methods:**

The temperature change in the fingertips before and after the application of COT in the corresponding hand was studied in healthy adult volunteers, donors after donating 400 mL of blood and in victims with blood loss of less than or more than 35%.

**Results:**

During COT, the temperature in the fingers of the ischemic hand decreased in all the subjects. After COT the temperature in the fingers rose above the baseline level in healthy volunteers and in donors who donated 400 mL of blood, but did not increase in most patients with massive blood loss, of which some patients died despite the treatment.

**Conclusions:**

We report the dynamics of local temperature in the finger pads after the COT in healthy adult volunteers, in donors after they donated 400 mL of venous blood each, and in victims with massive blood loss less than or greater than 35%. It is shown that the detection of local hyperthermia in the finger pads after occlusion is a sign of good adaptation to hypoxia and the probability of survivability of the victim with massive blood loss.

## 1. Introduction

The emergence of victims with massive blood loss is still highly probable in any country in the world, not only because of the probability of the use of firearms or knives by police and military personnel, but also because of the probability of various man-made disasters, car and other transport accidents in any part of the world [1, 2, 3, 4]. Therefore, in every populated area of the planet there is a 24-hour system of emergency medical care, designed, among other things, to provide urgent medical care to victims of massive blood loss [5]. The fact that massive blood loss can cause hemorrhagic shock, which often in a very short time leads to severe hypoxia and death of the victims from hypoxic damage to the brain cells [6].

In this regard, an accurate assessment of the severity of the victims’ health condition is very important in case of massive blood loss [7]. Traditional assessment of the severity of blood loss is based on the calculation of the volume of blood lost by the victim [1]. Currently, methods such as laser Doppler flowmetry and photoplethysmography are used to assess blood volume and blood flow [8, 9, 10]. The modern classification of blood loss is based on the relationship of calculated blood loss values to such clinical indicators as pulse rate, blood pressure, respiratory rate, diuresis rate and mental state [11]. At the same time, the use of this classification to assess the severity of blood loss in clinical practice has known limitations and drawbacks [12]. It is reported that in order to optimize the diagnosis, a method of interpretable machine learning retrospective analysis of large data from studies of patients with blood loss has been used [13]. The results give some hope to improve the prediction of hemorrhagic shock risk, but only in a certain group of patients. At the same time, the obtained results have not yet been clinically confirmed. But the mortality rate of victims of massive blood loss remains high [3].

One of the ways to reduce mortality during blood loss may be to increase the speed and accuracy of assessing the severity of blood loss for the health of victims by shifting the focus of diagnosis from assessing the amount of blood loss to assessing the ability of victims to adapt to it and to hypoxia [6].

Since blood vessels play a significant role in human adaptation to blood loss and hypoxia [14], one of the ways to improve the accuracy of blood loss severity assessment could be the assessment of vascular reactivity in response to dosed blood loss and/or hypoxia. In this case, it is very likely that the adaptive mechanism may involve spasm of the blood vessels of the skin, the purpose of which is a consistent redistribution of oxygen delivery from the skin to the brain [15, 16]. At the same time, a change in local blood flow leads to a change in local temperature [17]. This relationship makes it possible to assess the intensity of peripheral blood flow (and reactivity of blood vessels) using infrared thermography [15, 17, 18]. In addition, when assessing blood perfusion, the thermography method has significant advantages over the methods used, in particular, the use of a thermal imager allows assessing blood perfusion by a non-contact method with high spatial resolution [19]. As an imaging technique, the value of modern infrared thermography is its ability to produce a digitized image or high speed video rendering a thermal map of the scene in false colour. The fact is that temperature is the most important condition for human metabolism and function. Therefore, thermal non-contact recording of temperature dynamics of exposed body parts is of great importance for assessing the condition of the victim, especially in military field conditions [20, 21].

The cuff occlusion test (COT) is one of the most well-known and traditional diagnostic tests of the cardiovascular system assessment using infrared thermography [22]. Based on this, the aim of the work was infrared monitoring of the temperature of the fingers during and after COT in adult healthy volunteers, as well as in adults with different amounts of blood loss.

## 2. Methods

We studied dynamics of fingertips temperature in 20 healthy volunteers (group 1), in 5 healthy blood donors (group 2) and in 35 patients treated in the department of anesthesiology and intensive care with the diagnosis: traumatic hemorrhagic shock (group 3). The dynamics of the local temperature was recorded in the pad of the finger having the longest length [22]. The exclusion criterion was the presence of Raynaud’s phenomenon, scleroderma, diabetes mellitus, alcoholism, drug addiction, COVID-19 in the studied. The diagnosis of hemorrhagic shock was based on Advanced Traima Life Support (ATLS) system.We assessed dynamics of temperature in patients admitted to the clinic with blood loss less than 35% of estimated amount of circulating blood (II class of blood loss according to ATLS, n = 21) (this is group 3a) and more than 35% (III–IV class of blood loss according to ATLS, n = 14) (this is group 3b). All patients under study underwent shortened cuff occlusion test. For this purpose the examinee was laid horizontally on the back, the cuff was applied to the shoulder area of “working” arm, inflated to the value exceeding systolic pressure by 30 mm Hg and kept this pressure for 2 minutes [22]. Infrared thermal images of the palm and palm surface of the fingers of the subjects were recorded before, during, and after the COT at a time interval of 30 seconds. Infrared temperature monitoring of selected body areas was performed using a ThermoTracer TH9100XX (NEC, USA). The ambient temperature in the investigated room was 24– ${\mathrm{25}}^{\circ }$C, the temperature window of the thermal imaging camera was set in the range from +25 to +36 ^∘^C. The obtained data were processed using Thermography Explorerand Image Processor software (Version: 4.7. GORATEC Engineering Gmbh, Germany). Quantitative data were presented as arithmetic mean (M), standard deviation (SD). One-way analysis of variance (ANOVA) was used to determine statistically significant differences between groups. The study protocol complied with the principles outlined in the Declaration of Helsinki of the World Health Organization, and was approved by the Ethics Committees at Izhevsk State Medical Academy (protocol number 477, April 16, 2016). All study subjects signed an informed citizen’s consent to participate in the study voluntarily.

## 3. Results

We conducted a study of the local temperature in the fingertips of adult men and women, whose age in the control group had no significant differences from the age of the studied donors and victims of blood loss. The characteristics of the composition of the subjects studied in 3 groups are presented in Table 1.

**Table 1.** **Baseline statistical characteristics of the study groups**.

Characteristics	Group 1 (n = 20)	Group 2 (n = 5)	Group 3 (n = 35)
Age, mean ± SD (years)	36 ± 11.5	42 ± 9.0	46 ± 12.5
Gender, %female	9/20 (45)	5/0 (0)	11/35 (30)
BMI, mean ± SD (kg/$m^{2}$)	27.1 ± 2.4	28.4 ± 1.2	28.2 ± 2.0

BMI, body mass index; SD, standard deviation.

In all groups of subjects 4 temperature marks were recorded: T0—initial temperature (before COT), T1—temperature 120 seconds COT, T90—temperature 90 seconds after COT, T300—temperature 300 seconds after COT. Initially, local temperature in the fingertips of all 60 subjects was examined immediately before the application of COT. The results obtained are shown in Table 2.

**Table 2.** **The temperature values of the fingertips of the examined before, during and after the COT**.

Temperature, mean ± SD (Max-Min), ^∘^C				
Study groups	T0	T1	T90	T300
Group 1	32.3 ± 3.8 (35.4–27.1)	29.5 ± 0.5 (33.6–26.3)	33.2 ± 3.7 (35.9–30.4)	32.1 ± 2.3 (35.3–28.0)
Group 2	32.0 ± 0.3 (34.0–26.6)	28.8 ± 0.6 (30.4–26.6)	33.2 ± 1.1 (35.7–30.2)	32.0 ± 0.2 (34.1–29.3)
Group 3a	27.3 ± 1.2 (30.6–25.0)	25.1 ± 0.1 (25.8–25.0)	26.2 ± 0.1 (29.3–25.0)	27.1 ± 1.0 (29.8–25.0)
Group 3b	26.3 ± 0.6 (28.0–25.0)	25.0 ± 0.0 (25.6–25.0)	25.5 ± 0.1 (27.0–25.0)	25.7 ± 0.2 (27.0–25.0)

COT, cuff occlusion test; SD, standard deviation.

The results showed that in all groups under study there was a change in local temperature as a result of the manipulations compared to the initial values. The values of the temperature difference (ΔT) are presented in Table 3.

**Table 3.** **The temperature difference values of the fingertips of the examined during and after the COT**.

ΔT (mean ± SD),^∘^C			
Study groups	ΔT1	ΔT90	ΔT300
Group 1	–2.7 ± 1.6	0.9 ± 0.6	–0.2 ± 0.2
Group 2	–3.3 ± 0.9	1.2 ± 0.5	0.2
Group 3a	–2.1 ± 1.3	–1.1 ± 0.7	–0.2 ± 0.7
Group 3b	–1.2 ± 0.7	–0.8 ± 0.5	–0.6 ± 0.5

COT, cuff occlusion test; SD, standard deviation; ΔT1 = T1–T0; ΔT90 = T90–T0; ΔT300 = T300–T0.

The effect of blood loss on the temperature difference was determined by F-tests in one-way ANOVA (Table 4), which showed that statistically significant differences between the groups were observed 90 seconds after ischemia elimination (ΔT90).

**Table 4.** **The value of the Fisher criterion at the level of significance α = 0.05**.

ΔT	ΔT1	ΔT90	ΔT300
F-value	0.3	5.4	0.3
F-critical	2.8	2.8	2.8

Our multiple comparisons (*t*-test between group 1 and group 2, group 1 and group 3a, group 1 and group 3b) with Bonferroni correction (0.017) confirmed the reliability of the identified differences.

The obtained data indicate that infrared thermography provides an accurate registration of the local temperature in the finger pads of all the examined subjects. However, the absolute values of local temperatures in the fingertips of individual examinees from different groups cannot be used as a measure of the reactivity of blood vessels in response to blood loss, since the temperature values were in the range of +27 –+30 ^∘^C in the examinees of all groups. In this connection, absolute values of local temperature in the fingertips measured by the traditional technology have no prognostic and diagnostic value.

We then studied the dynamics of local temperature in the fingertips during and after the application of COT. The results showed a high diagnostic and prognostic value of the dynamics of local temperature in the fingertips when COT was applied. It appeared that the dynamics of local temperature in the finger pads after COT depends on the degree of blood loss, reflects the magnitude of the reserves of adaptation to severe hypoxia and indicates the survival rate in this case.

**Fig. 1.**
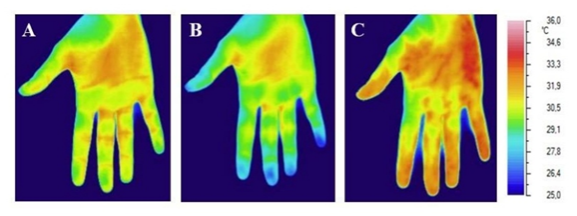
**Infrared image of the palmar surface of the right hand in healthy volunteer**. (A) Before the COT. (B) Immediately after the 2-minute COT. (C) After 90 seconds after the 2-minute COT.

Thus, in the control group (healthy volunteers) COT led to a decrease in local temperature in the finger pads, which decreased to +29.5 ± 0.5 ^∘^C by the end of COT. Then, after blood circulation was restored in the hand, the temperature in the finger pads began to increase and reached its maximum value after 90 seconds. The maximum temperature reached +33.2 ± 3.7 ^∘^C, which was 0.9 ± 0.6 ^∘^C higher than the temperature of the fingertips before COT. After that, the temperature in the fingertips slowly decreased and reached the initial values 300 seconds after the end of COT. The corresponding images on the thermal imager screen of the palm surface of the hand in a healthy volunteer are shown in Fig. 1.

From the above illustration, it is clearly seen that the ischemia of the hand created by COT is manifested by a decrease in local temperature. It can be seen that the pads of the fingers are cooled the most (their surface looks blue on the thermal imager screen). It is also clearly visible that 90 minutes after the ischemia of the hand (after the termination of COT), the palm surface of the hand becomes warmer than before the use of COT (in particular, the distal phalanges of the fingers look red on the thermal imager screen).

In the donor group, it was found that donating 400 mL of venous blood each led to a decrease in temperature in the fingertips from +33.6 ± 2.8 to +32.0 ± 0.3 ^∘^C. Then, immediately after the completion of venous blood donation, acute ischemia of the hand was created in the donors using COT. It was found that by the end of COT (i.e., after 2 minutes of hand ischemia), the temperature in the finger pads decreased from +32.0 ± 0.3 to +28.8 ± 0.6 ^∘^C. Then, after COT was stopped, the temperature in the fingertips began to increase and reached its maximum high value after 90 seconds. At the same time, the temperature in the finger pads exceeded the initial value by an average of 1.2 ± 0.5 ^∘^C. Thereafter, the temperature in the finger pads began to slowly decrease and reached baseline values after 300 seconds.

**Fig. 2.**
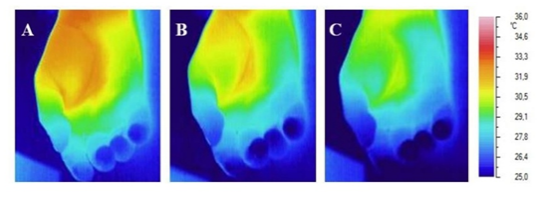
**Infrared image of the palmar surface of the right hand of the patient with hemorrhagic shock (blood loss >35%)**. (A) Before the COT. (B) Immediately after the 2-minute COT. (C) After 90 seconds after the 2-minute COT.

In the group of blood loss victims, the dynamics of local temperature in the finger pads after COT was different and depended on the volume of blood lost. It was found that in all the patients studied with massive blood loss after COT the local temperature in the finger pads did not exceed the baseline values. In subjects with blood loss <35% (group 3a) the temperature in the finger pads reached baseline values 300 seconds after COT and was +27.1 ± 1.0 ^∘^C, and in subjects with blood loss >35% (group 3b) 300 seconds after COT the temperature in the finger pads remained below baseline values and was +25.7 ± 0.2 ^∘^C. The corresponding images on the thermal imager screen of the palm surface of the hand of a patient with massive blood loss are shown in Fig. 2.

The illustration clearly shows that after massive blood loss, the use of COT does not significantly change the dynamics of local temperature in the hand and fingers. It is clearly visible that on the thermal imager screen, the fingertips look blue before, during and after the COT. Especially clearly visible is the absence of redness in the fingertips after the COT.

The results of hospital treatment of patients in the anesthesiology and intensive care unit showed that all patients with blood loss, in whom the temperature of the finger pads reached baseline values by the 5th minute, survived. Of the 14 patients whose temperature remained below baseline values after cessation of ischemia, 4 patients died within 48–72 hours despite standard treatment. The degree of their blood loss corresponded to ATLS class IV blood loss and amounted to more than 60% of estimated circulating blood volume.

## 4. Discussion

Massive bleeding is still one of the predominant causes of death among patients with potentially treatable injuries [3, 4]. The reason for the high mortality rate, in our opinion, is the lack of a method for timely and accurate assessment of the victim’s adaptive capacity to hypoxia [6, 22]. The fact is that the true cause of death in hemorrhagic shock is hypoxic brain damage. At the same time, the danger of massive hemorrhage is still estimated by the volume and severity of blood loss, rather than by the severity of hypoxia and the reserves of adaptation to it [23].

In our work, we assumed that the leading role in human adaptation to hypoxia is played by peripheral blood vessels, the spasm of which provides centralization of blood circulation. Therefore, it was suggested that one of the ways to improve the accuracy of the assessment of the system of adaptation to hypoxia could be the assessment of vascular reactivity in response to dosed hypoxia and/or blood loss. In turn, the assessment of peripheral circulation and microcirculation under hypoxia can be mediated by the dynamics of local surface temperature of the exposed body part, for example in fingertips [18]. It was assumed that spasm of blood vessels in the skin of the fingers reduces the inflow of warm arterial blood to them, so when surrounded by air with a temperature of +24–+26 ^∘^C, it can lead to cooling of the fingers.

Further, we assumed that there is no alternative to infrared thermography to record the dynamics of local finger temperature under hypoxia and blood loss, and there is no alternative to cuff occlusion test to assess cardiovascular reactivity. Therefore, our study was carried out with thermal imaging and COT. The obtained results confirmed the correctness of the assumptions.

It turned out that COT causes a temperature drop in the fingertips of absolutely all adult healthy volunteers. Then, immediately after COT is stopped (after the ischemia in the hand is eliminated and blood flow is restored), the cooled fingers begin to warm up quickly. The temperature in the fingers rises above the initial values.

Similar development of local hyperthermia was detected in donors immediately after they donated 400 mL of venous blood.

In this regard, local hyperthermia in the fingers, developing after COT and safe blood loss, was considered by us as a normal adaptive vascular reaction in response to safe ischemia and blood loss. Therefore, local hyperthermia in the fingertips after COT may be a diagnostic symptom of the good adaptive reserves to hypoxia after blood loss and a prognostic symptom of survival.

Thereafter, we investigated the dynamics of temperature in the fingertips after COT in patients with massive blood loss. It turned out that the majority of victims with blood loss of less than 35% had no reactive hyperthermia in the finger pads after COT (in 15 of 21 subjects), and the minority (6 of 21 subjects) had weakly pronounced reactive local hyperthermia. Because that we can conclude that the reserves of adaptation to hypoxia were exhausted.

In 13 of the 14 victims with blood loss of more than 35%, the use of COT led to a decrease in temperature in the finger pads for a long period of time without a subsequent phase of reactive hyperthermia in them. At the same time, the clinical condition of these patients was extremely severe. This suggests that in 13 out of 14 patients with blood loss exceeding 35%, the reserves of adaptation to acute hypoxia were exhausted. This conclusion was confirmed by the worst clinical results of treatment and the death of 4 patients despite the treatment.

**Fig. 3.**
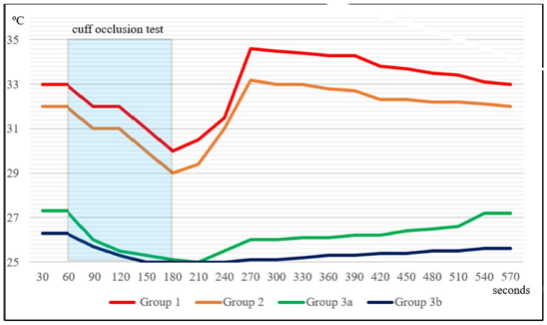
**Summary graph of the temperature dynamics in the fingertips after the cuff occlusion test in healthy adults and in adults after they lose different volumes of blood**. In group 1 and group 2 (normal and safe blood loss, respectively) local hyperthermia develops after COT, in group 3 and group 4 (high and extremely high life-threatening blood loss, respectively) no local hyperthermia develops after COT.

A summary graph of the temperature dynamics in the fingertips after the cuff occlusion test in healthy adults and in adults after they lose different volumes of blood is shown in Fig. 3.

The illustration clearly shows that COT reduces the temperature in the fingertips of the hand regardless of bleeding and the amount of blood lost. However, after COT, the dynamics of temperature in the fingers of healthy people and those affected with massive blood loss are different. It can be seen that in volunteers without blood loss and in donors after they donate 400 mL of blood, the temperature in the fingers begins to rise immediately after the termination of COT and rises very quickly above the initial values. At the same time, in patients with massive blood loss, the temperature in the fingers remains low for a long time, then gradually begins to rise, but does not reach the initial temperature level.

Despite the fact that we followed the recommendations for thermal imaging measurements [18], in our studies we were forced to agree that the victims with blood loss had an initial temperature different from the initial temperature in healthy volunteers. It should be noted that the basal temperature difference between the 1st and 2nd groups has no significant differences. Since the sample size (especially for group 2) was small (5 people), the results obtained by us require additional confirmation in the future on a larger number of donors. At the same time, the different initial temperature in group 3 compared to the initial temperature in group 1 may be caused by pathology. In particular, the difference may be explained by the development of vascular spasm in people in group 3. This vascular spasm in the fingertips may be part of adaptive mechanisms aimed at centralizing blood circulation [24, 25].

The results showed that infrared monitoring of fingertip temperature after COT allows real-time assessment of the magnitude of adaptive reserves to hypoxia after blood loss. In particular, postocclusive reactive hyperthermia in the fingertips indicates a high probability of patient survival despite blood loss. The developed technology and the method of thermal imaging are easily applicable in clinical practice. It is likely that an automated approach should be preferred for implementing the developed method in clinical practice. In particular, machine learning experience gained from the use of thermal imaging for assessing vascular diseases in clinical practice can be used for this purpose [26].

A probable mechanism for the disappearance of reactive local hyperthermia in the fingertips of the patients studied with blood loss may be the development of microvascular endothelial dysfunction [27, 28]. Endothelial cells are known to regulate vasoreactivity and vascular permeability through the synthesis of nitric oxide, a powerful vasodilator [29]. In severe hemorrhagic shock and trauma, the endothelium is damaged, resulting in endothelial dysfunction [30]. Obviously, more research is needed to confirm these assumptions. In addition, our studies did not take into account the level of hemoglobin and hematocrit in the subjects in all groups. It is quite possible that such studies will help supplement the informativeness of the developed method in the future.

## 5. Conclusions

(1) The dynamics of local temperature in the fingertips after cuff occlusion test can be a simple way to assess the reserves of adaptation to hypoxia after blood loss.

(2) Post-occlusive reactive local hyperthermia in the fingertips indicates good resistance to hypoxia caused by blood loss and indicates a high probability of survival of the patient despite blood loss.

(3) The absence of reactive hyperthermia after cuff occlusion test may be a sign of exhaustion of vascular adaptation reserves to hypoxia and a prognostic sign of low survival in massive blood loss.
